# The Exoproteome of *Staphylococcus pasteuri* Isolated from Cervical Mucus during the Estrus Phase in Water Buffalo (*Bubalus bubalis*)

**DOI:** 10.3390/biom12030450

**Published:** 2022-03-15

**Authors:** Mahalingam Srinivasan, Subramanian Muthukumar, Durairaj Rajesh, Vinod Kumar, Rajamanickam Rajakumar, Mohammad Abdulkader Akbarsha, Balázs Gulyás, Parasuraman Padmanabhan, Govindaraju Archunan

**Affiliations:** 1 Department of Animal Science, Bharathidasan University, Tiruchiraappalli 620024, India; srinivasanma@bdu.ac.in (M.S.); r.durairaj@group-irsea.com (D.R.); 2 Animal Physiology Division, National Institute of Animal Nutrition and Physiology, Bangalore 560030, India; 3 Department of Biotechnology, School of Chemical and Biotechnology, SASTRA Deemed University, Thanjavur 613401, India; sasmuthu@gmail.com or; 4 Research Institute in Semiochemistry and Applied Ethology, 84400 Apt, France; 5 Laboratory for the Conservation of Endangered Species, CSIR-Centre for Cellular and Molecular Biology, Uppal Road, Hyderabad 500007, India; vinod@ccmb.res.in; 6 Department of Biotechnology, Marudupandiyar College, Thanjavur 613403, India; biotechrajakumar@gmail.com; 7 Mahatma Gandhi-Doerenkamp Center, Bharathidasan University, Tiruchirappalli 620024, India; akbarbdu@gmail.com; 8 Research Co-Ordinator & Department of Biotechnology, National College (Autonomous), Tiruchirappalli 620001, India; 9 Lee Kong Chian School of Medicine, Nanyang Technological University, Singapore 636921, Singapore; balazs.gulyas@ntu.edu.sg; 10 Dean of Research, Marudupandiyar College, Thanjavur 613403, India

**Keywords:** extracellular proteins, enolase, cpn60, lipoprotein, chemical communication, microbes

## Abstract

Bacterial extracellular proteins participate in the host cell communication by virtue of the modulation of pathogenicity, commensalism and mutualism. Studies on the microbiome of cervical mucus of the water buffalo (*Bubalus bubalis*) have shown the occurrence of *Staphylococcus pasteuri* and that the presence of this bacterium is indicative of various physiological and reproductive states in the host. Recently, *S. pasteuri* has been isolated from the cervical mucus of the buffalo during the different phases of estrous cycle, and has proved to be much more pronounced during the estrus phase. The basis underlying the availability of a significantly increased *S. pasteuri* population, specifically during the estrus phase, is not known. Consequently, it is important to determine the significance of the specific abundance of *S. pasteuri* during the estrus phase of the buffalo host, particularly from the perspective of whether this bacterial species is capable of contributing to sexual communication via its extracellular proteins and volatiles. Therefore, the relevance of *S. pasteuri* exoproteome in the buffalo cervical mucus during the estrus phase was analyzed using LC-MS/MS. As many as 219 proteins were identified, among which elongation factor Tu (EF-Tu), 60-kDa chaperonin (Cpn60), enolase, fructose-bisphosphate aldolase class 1 (FBP aldolase), enoyl-[acyl-carrier-protein] reductase [NADPH] (ENR) and lipoprotein (Lpp) were the functionally important candidates. Most of the proteins present in the exoproteome of *S. pasteuri* were those involved in cellular–metabolic functions, as well as catalytic- and binding activities. Moreover, computational studies of Lpp have shown enhanced interaction with volatiles such as acetic-, butanoic-, isovaleric- and valeric acids, which were identified in the cervical mucus *S*. *pasteuri* culture supernatant. The present findings suggest that *S. pasteuri* extracellular proteins may play an important role in buffalo sexual communication during the estrus phase.

## 1. Introduction

The bacterial exoproteome is the major medium of communication between bacteria and the host in regard to pathogenicity, commensalism and mutualism. Especially, the extracellular proteins of bacteria are involved in several biological processes such as nutrient recognition, binding, degradation, permeability of extracellular molecules, cellular communication, environmental detoxification of environment, attachment to the host cell, transduction of signals, virulence and immune recognition [1,2]. Extracellular proteins account for 10–30% of the proteins encoded by respective bacterial genomes [3,4]. In the human gut, commensal bacteria co-evolved with their hosts by molecular association to engage in adhesion, epithelial barrier activity and immune system modulation [5,6]. Bacterial communities in the vaginal microbiome plays important roles during various aspects of reproduction such as fertilization, maintenance of fetuses, delivery, etc. Yet, there are only very limited reports about the composition of microbiome during different phases of the estrous cycle in cows [7] and buffaloes [8].

Also, the variety of bacteria present during the different phases of the estrous cycle of mammals may signify some essential role in chemical communication. A recent study has confirmed the abundance of bacteria belonging to phylum Firmicutes in buffalo cervical mucus (CM) during the estrus phase compared to the other phases [8]. Further, a culture-dependent study showed a specific expression of *S. pasteuri* during the estrus phase in buffalo CM. It is therefore evident that *S. pasteuri* may have a significant role in the production of volatiles such as acetic-, butanoic, propanoic, isobutyric-, valeric, and isovaleric acids in buffalo [9]. Further, behavioural assessments have shown that the volatiles facilitate attraction towards the opposite sex and encourage engaging in the mating stance in buffaloes [10,11]. Moreover, the immune system undergoes greater challenges during the estrus phase, when the cervix is open, providing opportunity for vaginal bacteria spread toward the cervix and the uterus. During estrus, estrogen levels have a great impact on CM characteristics such as a reduced viscosity and the development of an alkaline pH compared to the other phases of the estrous cycle [12].

A vast majority of bacterial lipoproteins (Lpp) are derived from fatty acids; however, each species of bacteria has unique Lpp, based on its genetic makeup and habitat [13]. The specific role of Lpp in bacteria is still elusive. The roles of about 30% of the Lpp are not yet known. Therefore, the functional significance of Lpp is unclear, and studies are warranted to expound whether Lpp has any role in immune surveillance or proinflammatory/immune modulating signals. Moreover, the bacterial lipoproteins belong to the lipocalin family, among which Blc from *E. coli* has been elaborately studied [14]. Lpp has a type 2 signal peptide which allows for periplasm to be exported and leads to anchoring in the inner leaflet of the outer cell membrane [15,16]. It is well established that lipocalins bind with many small hydrophobic volatiles for chemical communication. Also, the reported buffalo nasal odorant binding proteins belong to the lipocalin family which plays a significant role in the perception of the pheromones and/or volatiles [17,18].

Protein biomarkers are great tools with a wide range of applications in the development of clinical and biomedical diagnostics. In this regard, it is important to explore the extracellular proteins of bacteria which inhabit the vaginal mucus, and investigate their role in pheromone communication during the estrus phase. Therefore, the present study was undertaken to establish the extracellular proteins profile of *S. pasteuri* using high resolution liquid chromatography-tandem mass spectrometry (LC-MS/MS) and to analyze the interactions of Lpp with the reported volatiles and/or pheromones using in silico approaches. Ultimately, the findings open up a new window in the study of bacterial exoproteome for chemical communication in mammals.

## 2. Materials and Methods

### 2.1. Bacterial Strain and Protein Isolation

For the isolation of bacteria, the CM was collected from estrus buffaloes as described in the Supplementary File S1 (S2.1) [9]. The estrus-specific *S. pasteuri* strain BCVME2 was cultured in mannitol salt agar [19]. The plates were incubated at 37 °C for 24–48 h [9]. Pure bacterial culture was inoculated with 0.5 L of tryptic soy broth (TSB) in order to obtain the extracellular proteins. For plotting the growth curve (Supplementary File S1 Figure S1), bacterial cultures were measured at OD_600_ for every 30 min in a PC-Based Double Beam UV-VIS Spectrophotometer (Systronics, Bangalore, KA, India). When the bacteria reached the stationary phase (6 h), the cultures supernatants were obtained by centrifugation for 30 min at 5000× *g* at 4 °C and further processed for protein extraction [16].

### 2.2. Protein Precipitation and Estimation

The trichloroacetic acid (TCA)-acetone procedure was followed to precipitate the bacterial extracellular proteins [20]. Briefly, the samples were incubated overnight at −20 °C with equal volume of 20% TCA. After incubation, the samples were centrifuged at 5000× *g* at 4 °C for 30 min. The pellets were washed three times with ice-cold acetone and centrifuged at 5000× *g* at 4 °C for 5 min. The pellets were air-dried and re-suspended in the 50 mM Tris-HCl (pH-8). The protein concentration was determined by a modified Bradford method [21].

### 2.3. SDS-PAGE Analysis

The precipitated protein sample was mixed with 2× Laemmli electrophoresis buffer and loaded on 12% SDS-PAGE gels [22]. The molecular range of protein profiles was identified by running molecular mass reference standards (Bio-Rad, Hercules, CA, USA). The electrophoresis was carried out at 37 °C for 4 h, initially at 50 V for the stacking gel and then at a constant 100 V for the separating gel. On completion of electrophoresis, the gel was rinsed with distilled water and stained with 0.5% Coomassie brilliant blue R-250 prepared in 40% methanol and 10% acetic acid. The gel was then de-stained in a solution containing 40% methanol and 10% acetic acid until the protein bands were visible.

### 2.4. Trypsin Digestion

Bacterial culture supernatant protein bands from SDS-PAGE were trimmed out, and then reduced with 10 mM dithiothreitol (DTT) in 50 mM ammonium bicarbonate at 56 °C for 45 min. Subsequently, the SDS gel fragments were alkylated with 55 mM iodoacetamide in 50 mM ammonium bicarbonate at 37 °C for 30 min. In-gel digestion was performed for the de-stained SDS-PAGE using the standardized protocol [23] with sequencing grade trypsin (Roche, Risch-Rotkreuz, Switzerland). Desalting of peptides was performed using Pierce^®^ C18 tips before loading in Nanospray capillary column (PepMap^TM^ RSLC C18, Thermo Fisher Scientific, Waltham, MA, USA).

### 2.5. Liquid Chromatography-Tandem Mass Spectrometry

The digested peptides were subjected to LC-MS/MS in a Q-Exactive HF mass spectrometry (Thermo Fisher Scientific, Waltham, MA, USA). The conditions of LC-MS/MS are furnished in the Supplementary File S2. The protein spectra thus obtained were analyzed using the software Proteome Discoverer Version 2.2.0.388 (Thermo Fisher Scientific, Waltham, MA, USA) with the SEQUEST HT engine. The MS/MS spectra of the peptides were analyzed against a Universal Protein Resource (UniProt) database organism: *Staphylococcus pasteuri*; enzyme: trypsin; search parameters for identification of sequences were a precursor mass tolerance of 5 ppm and a fragment mass tolerance of 0.05 Da. The target FDR (strict) and target FDR (relaxed) were set at 0.01, and the validation was based on the q-value. Peptides sequenced by MS/MS were searched and highlighted on the aligned protein sequences using a macro code snippet. The profiled protein mass spectrometric data have been deposited at the ProteomeXchange database (Accession number: PXD023616).

### 2.6. Comparative Gene Ontology 

The total extracellular proteins of *S. pasteuri* were segregated based on biological processes, cellular components and molecular functions. The gene ontology (GO) map was developed using a web-based GO analysis tool ComparativeGO (https://www.comparativego.com/) (accessed on 13 December 2020) [24].

### 2.7. Protein Selection for Modelling 

The lipoprotein (Lpp) was selected from *S. pasteuri* extracellular protein profile and the protein sequence was retrieved from Universal Protein Resource (UniProt) (http://www.uniprot.org) (accessed on 5 January 2021) and its accession ID is A0A0M2NSM8. This protein is reported as a membrane-associated protein, which plays an anchoring role during signal transduction. Further, the sequence was cross-checked with the NCBI database and found to be related to the MetQ/NIpA family ABC transporter substrate-binding protein.

### 2.8. Physicochemical Properties and Secondary Structure Prediction of Lpp

The physicochemical properties of Lpp were analyzed using the ProtParam tool (https://web.expasy.org/protparam/) (accessed on 5 January 2021) in the ExPASy Bioinformatics Resource Portal [25]. The tool was used to compute the molecular weight (MW), amino acid composition (AA), theoretical isoelectric point (pI), extinction coefficient (EC), instability index (II), aliphatic index (AI) and grand average of hydropathy (GRAVY) of lipoprotein (Lpp). The molecular weight and theoretical pI were calculated as in Compute pI/MW. SOPMA web servers were used to predict secondary structure (2D) components [26]. The percentages of secondary structure components were predicted based on analysis of the relative frequencies of each amino acid in the helices, sheets and turns present in the protein sequence. Further, the Lpp protein sequence was submitted to SOPMA, and the results were validated with the ProFunc tool (https://www.ebi.ac.uk/thornton-srv/databases/profunc/) (accessed on 22 January 2021), which also depicted the biochemical function, 2D structure (alpha helix, extended strand, Beta turn, random coil) and three dimensional (3D) structures.

### 2.9. Multiple Sequence Alignment and Phylogenetic Tree Construction

The Lpp sequence (UniProt accession ID: A0A0M2NSM8) was submitted to BLASTp (Basic Local Alignment Search Tool proteins)-PSI-BLAST (Position-Specific Iterated BLAST) against non-redundant protein sequence database in NCBI. The lipid binding protein sequence orthologs of the mammalian odorant binding proteins (OBP) were obtained from the NCBI and submitted for the multiple sequence alignments (MSA) using the Clustal Omega server. These sequences were used to construct the phylogenetic tree to find the evolutionary diversity and neighbour member between Lpp and OBP. The unaligned and gap regions were edited manually by review in ClustalW alignment file (.aln) using the Molecular Evolutionary Genetics Analysis (MEGA) V.6.0 software. MEGA software enables calculation of p-distance of aligned proteins residues and the final sequence alignment session (.mas) exported in MEGA (.meg) file. The accuracy of the phylogenetic distribution of branches was determined based on 1000 bootstraps (BS). The Lpp protein dataset was employed to construct a phylogenetic tree adopting maximum likelihood.

### 2.10. Homology Modelling, Structure Validation and Binding Site Analysis

The selected Lpp protein sequence was submitted to the BLASTp and PSI-BLAST interaction against the PDB protein database for the template identification [27]. The best hit template was identified using blast score and the sequence identity of 48.98% with the 4EF1 template (pheromone cOB1 precursor/lipoprotein from *Enterococcus faecalis* V583). The Lpp sequence was submitted to the homology modelling server SWISS-MODEL (https://swissmodel.expasy.org/) (accessed on 8 January 2021) and the lipoprotein homology model was obtained by using a suitable template consisting the crystal structure of the pheromone cOB1 precursor/lipoprotein (PDB ID: 4EF1_chain A_1.90 Å resolution). The Lpp protein model was built using the ProMod3 (Release: 3.2.0) tool viewed with the UCSF Chimera tool. The ProFunc tool was used to calculate the allowed and disallowed regions of the modelled protein. The allowed and additional allowed regions of residues were calculated using a Ramachandran plot. Further, the structure validation was conducted by the QMEAN Server.

The receptor model Lpp was submitted to the CASTp [(Computed Atlas of Surface Topography of protein server) (http://sts.bioe.uic.edu/castp) (accessed on 21 January 2021)] for identification of active sites on the protein surface. The server predicted the binding site by the alpha shape method, and the binding pockets were measured using the probe sphere 1.4 Å. Further, the structures were submitted to the DSV tool (Discovery Studio Visualizer), which facilitated the finding of the active site, XYZ grid points, for the docking analysis.

### 2.11. Molecular Docking Studies 

The molecular docking study was performed using Autodock [28], which is the most common means of performing receptor ligand docking. The docking program was run through the DockingServer (https://www.dockingserver.com) (accessed on 28 January 2021), which is a web-based graphical user interface module that consists of different charge calculation methods, to enhance the accuracy of docking output [29]. The docking simulation was performed using LGA (Lamarckian genetic algorithm) [30].

### 2.12. Protein Optimization and Grid Construction

The homology-modelled Lpp (.pdb file) was submitted to the DockingServer for the analysis of receptor-ligand interaction. The active sites of 23 × 23 × 23 (nx, ny and nz) were selected as the grid radius and the cx = −2.61, cy = −21.18 and cz = −6.38 were selected as grid box coordinates for the grid box selection in Lpp homology modelled protein. The protein model was optimized using the Gasteiger charge calculation method. All rotatable torsions were released during docking. Each docking calculation was derived from 100 different runs that were set to terminate after a maximum of 2,500,000 energy evaluations. The population size was set to 150. During the search, a translational step of 0.2 Å, and quaternion and torsion steps of 5 were applied. Furthermore, the residual interactions were analyzed between protein and ligands using UCSF Chimera, and DSV tool.

### 2.13. Ligand Collection and Optimization

The compounds/volatiles present in *S. pasteuri* exoproteome were identified, and the 3D structures of the reported compounds were collected from PubChem (https://pubchem.ncbi.nlm.nih.gov/) (accessed on 21 January 2021). Then, the ligands were optimized by Gasteiger charges such as protein optimization procedures. The selected ligands were tabulated with the physicochemical properties corresponding to the PubChem ID. The details about the ligand were included in the chemical formula, molecular weight (MW), and other physicochemical properties as shown in Supplementary File S1 Table S1. Further, the residual interactions of ligands with the Lpp were validated using DSV tool. These computational procedures have been followed to support the protein-ligand interaction and structural conformation stability.

## 3. Results

### 3.1. Total Exoproteome of S. pasteuri

**Figure 1:**
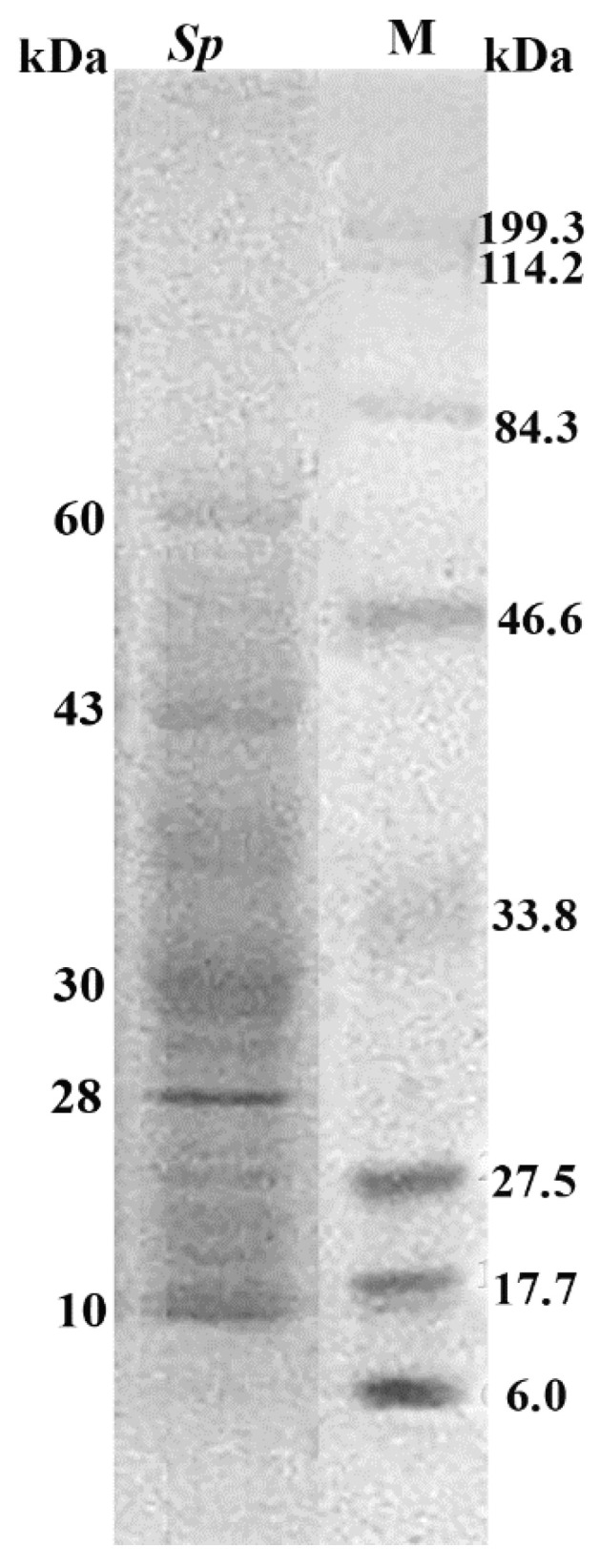
SDS-PAGE. Single dimensional gel electrophoresis of extracellular proteins of *S. pasteuri* isolated from estrus CM. kDa on the right denotes the standard protein molecular weight (MW) marker. kDa on the left shows the approximate MW of high intensity proteins from *S. pasteuri.* The original figure is provided in the Supplementary File S1 Figure S2 from which lane 5 and the marker lane are used here as the major figure for a better understanding of the exoproteome.

The exoproteome of *S. pasteuri* was analyzed in 12% SDS-PAGE (Supplementary File S1 Figure S2). The proteins ranged in MW from 80 to 10 kDa. The protein bands of MW 60, 43, 30, 28 and 10 kDa were prominent (Figure 1). 

### 3.2. Mass Spectrometry Analysis

A total of 219 proteins were identified in the *S. pasteuri* culture supernatant (Supplementary File S1 Table S2). Among them, the notable proteins (Table 1) were elongation factor Tu (EF-Tu), 60 kDa chaperonin (Cpn60), enolase, fructose-bisphosphate aldolase class 1 (FBP aldolase), enoyl-[acyl-carrier-protein] reductase [NADPH] (ENR) and lipoprotein (Lpp). The tandem mass spectra of identified Lpp peptide sequences and matched amino acids are represented (Supplementary File S1 Figure S3 and Supplementary File S3). Theoretical molecular weight of the Lpp is 30.1 kDa, which matches the expected molecular weight determined from the SDS-PAGE gel.

### 3.3. Comparative Gene Ontology

**Figure 2:**
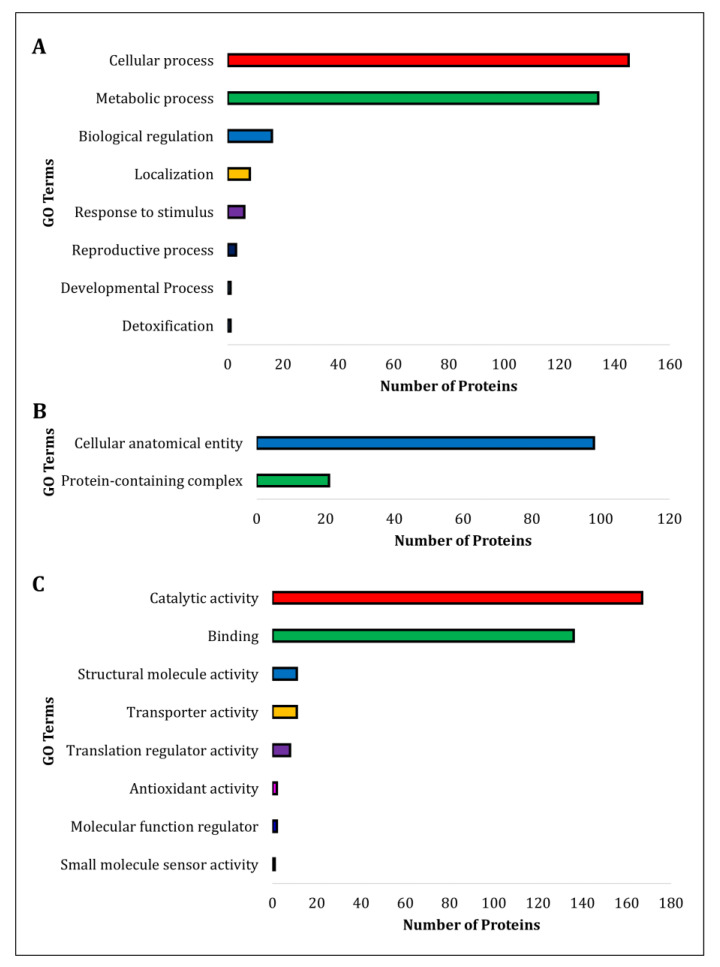
Functional annotation. The identified proteins from *S. pasteuri* culture supernatant were submitted to the ComparativeGO database, and the proteins were classified according to Biological process (**A**), Cellular component (**B**), and Molecular function (**C**). (**A**) Proteins were mainly involved in cellular and metabolic activities. (**B**) The majority of proteins were present in cellular anatomical entities and protein-containing complexes. (**C**) The major proteins were associated with catalytic and binding activities.

The proteins identified in the *S. pasteuri* culture supernatant were subjected to functional annotation. The protein ID’s (Uniprot Protein Accession Numbers) were extracted based on mass spectrometry data and executed in ComparativeGO for the gene ontology analysis. Among the 219 protein ID’s, only 209 were annotated in the server (Supplementary File S1 Figure S4). Further, the GO terms were classified based on their biological process, cellular components and molecular function (Figure 2A–C). The analysis revealed that the identified proteins were highly associated with catalytic and binding activities, followed by structural molecule-, transporter-, translation regulator-, antioxidant-, molecular function regulator-, and small-molecule sensor activities.

### 3.4. Properties of Lipoprotein

The ProtParam tool was employed to analyze the amino acid composition and physicochemical properties of lipoprotein sequences. It was revealed that the Lpp has 269 residues, and its molecular weight was 30.1 kDa. The theoretical pI was calculated as 9.27. The sequence has 49 positively charged (Arg and Lys combined) and 38 negatively charged (Asp and Glu combined) residues. The Grand hydropathicity average value (GRAVY) measured 0.570.

### 3.5. Secondary Structure Prediction

The SOPMA is a web-based homologue method as proposed by NPS@ (Network Protein Sequence Analysis) web server. This analysis revealed Lpp sequence having an extended strand (Ee) 16.36%, followed by random coil (Cc) 36.06% and alpha helix (Hh) 105%. The beta turn (Tt) 8.55% was least frequent (Supplementary File S1 Figure S5A). Representations of structural components are displayed in Lpp sequence (Supplementary File S1 Figure S5B).

### 3.6. Multiple Sequence Alignment

**Figure 3:**
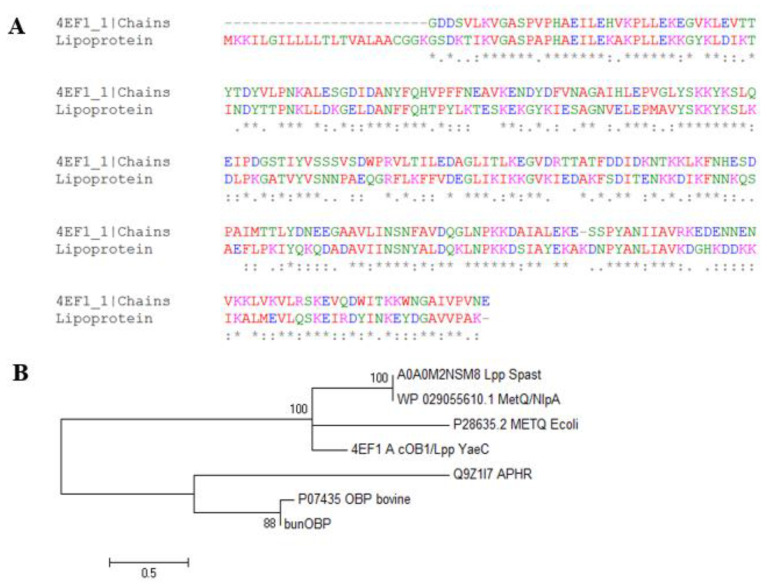
Multiple sequence alignment and phylogenetic tree of Lpp. (**A**). The alignment of Lpp sequence showed several identical residues predicted with the 4EF1. (**B**). The evolutionary tree represents clustering pattern of the selected Lpp, MetQ/NlpA and the OBP proteins.

The Lpp sequence was submitted to the ClustalO alignment tool, and aligned with pheromone cOB1 precursor/lipoprotein from *Enterococcus faecalis* V583, and the results showed the maximum number of identical residues, and also represented the most frequent conserved residues (Figure 3A). This protein shared 49% of its identity with Lpp sequences and was selected as a template. Several identical motifs with the pheromone precursor/lipoprotein were identified. Additionally, MetQ/NlpA family lipoprotein, bovine odorant binding protein (P07435 OBP), aphrodisin (Q9Z1l7 APHR), and buffalo nasal OBP (BunOBP) were subjected to phylogenetic analysis. This analysis revealed that Lpp has significant similarity with other lipoprotein sequences (4EF1) having regions of conserved identities and binding motifs. The MetQ/NlpA sequence was the neighbour member for Lpp. The bovine OBP and aphrodisin of hamster protein appeared in separate clusters.

The OBP and Lpp were compared with in order to identify the evolutionary relationships between proteins because both the proteins perform lipid binding and shuttle functions. The results showed that Met-ABC transport protein is a member closer to the Lpp of *S. pasteuri* (Figure 3B).

### 3.7. Homology Modelling and Validation

**Figure 4:**
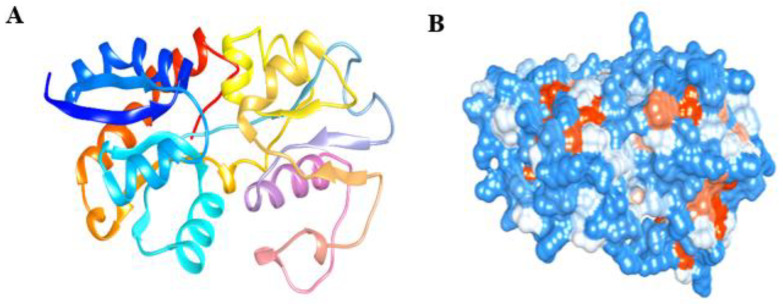
Homology model of Lpp protein. The protein is displayed by molecular 3D view with the representation of a rainbow color pattern from N-terminal to C-terminal (**A**), and the solid surface representation of Lpp as shown by hydrophobicity surface view (**B**), and it is best for showing the shape of the pocket. The surface view color pattern indicates most hydrophilic residues in blue, the strongest hydrophobic residues in orange red and the intermediate residues in white.

Aided by the validation, several similar sequences were observed in the same cluster where Lpp (A0A0M2NSM8) was reflected in the evolutionary analyses. The pheromone cOB1 precursor/lipoprotein is a membrane-segregated lipoprotein in *Enterococcus faecalis* [protein data bank (PDB): 4EF1] that shares the highest sequence identities with Lpp. The homology model of Lpp was constructed, and the structure showed an alpha-helix-linked-beta-strand with a compact surface for ligand binding. The structure was visualized using UCSF Chimera tool. The modelled Lpp structure is shown in different views such as molecular 3D view (Figure 4A), and solid surface view (Figure 4B). 

**Figure 5:**
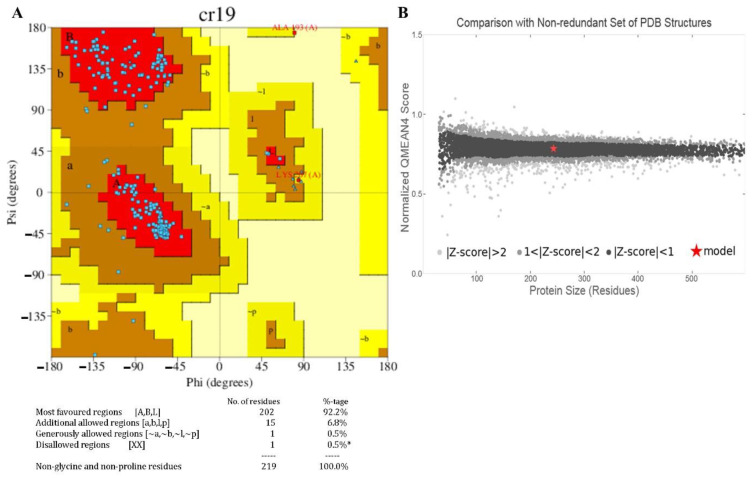
The validation of the homology modelled Lpp. The model was validated using Ramachandran plot analysis (**A**), the plot shows the most favoured and allowed residues in the limited phi/psi regions. “*” indicates that only few residues are present in the disallowed region of Ramachandran plot. The Z-score plot (**B**) shows the Lpp structure similar to native protein.

The beta-strands and alpha-helix were interconnected with the loops. The stereo-chemical quality of the 3D-model has been validated using ProFunc tool, and the phi-psi values of all amino acids were found to be present within the outer limit of Ramachandran map. The present structure validation results show that the residues of Lpp are laid in the most favoured (92.2%) and the additionally allowed regions (6.8%) in the Ramachandran plot. Additionally, the Z-score plot displayed a significant normalized global QMEAN score of 0.40 and per-residue model quality, and GMQE is 0.73 (accuracy of the tertiary structure) for the homology model (Figure 5A,B).

### 3.8. Binding Site Analysis

**Figure 6:**
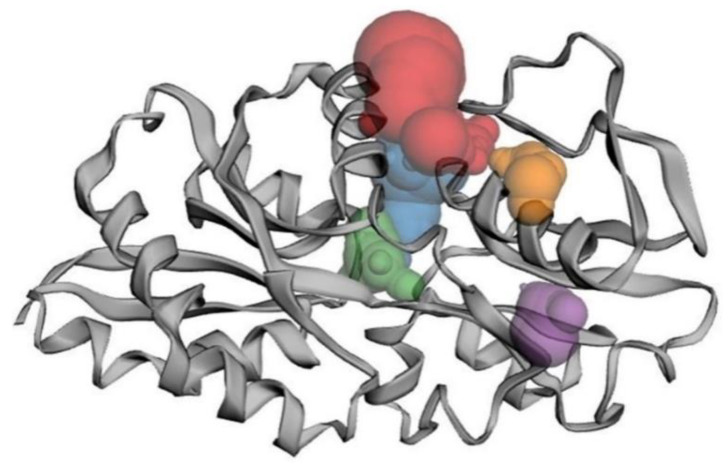
Binding cavity of Lpp. Hydrophobic residues present in the major and minor cavities of the protein model were visualized. The top 5 binding sites (red, blue, green, purple, orange) were observed using the CASTp server. The first red color-marked cavity shows the larger area and volume in the Lpp protein. Both area and volume shown are solvent-accessible surface area/volume (Lee-Richard molecular surface).

The potential binding sites and the binding residues of Lpp model were obtained from the CASTp 3.0 (Figure 6). The top five binding sites were colored differently to visualize the volume of the site, and these sites were predicted using alpha-shape method. The binding site of Lpp showed an area of 235.25 (A^2^) and a volume of 184.112 (A^3^).

### 3.9. Molecular Interaction of Lpp

**Figure 7:**
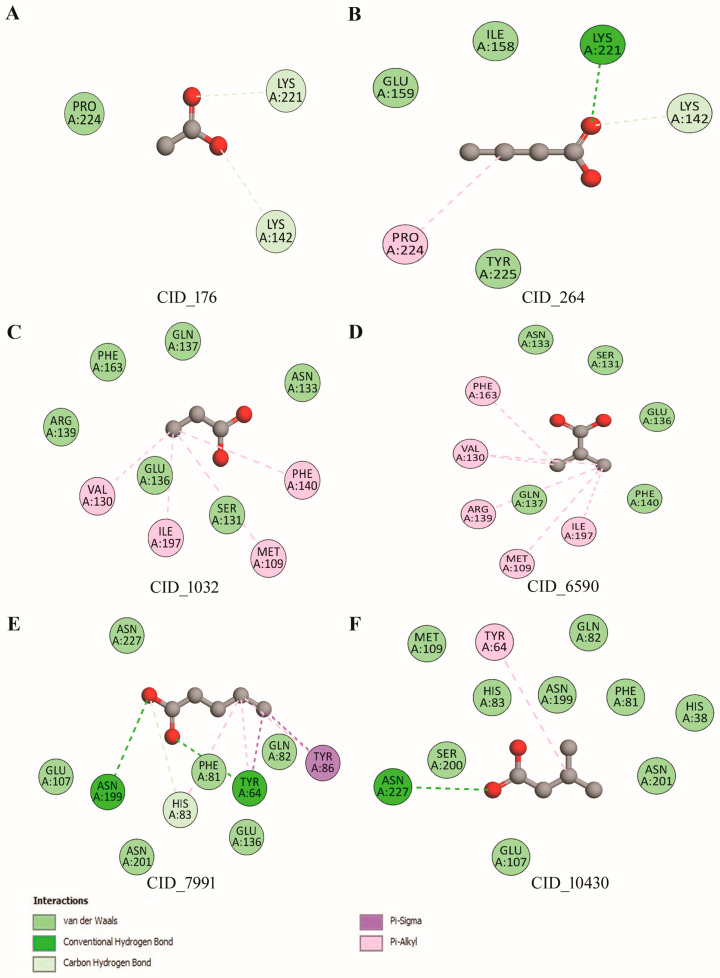
Interactions of the Lpp with the pheromones/volatiles. The 2D residue map of pheromone interactions with the Lpp is shown, and the residual interactions were observed below 4.0 Å. The acetic (CID_176) and butanoic acids (CID_264) have several H-bond interactions (**A**,**B**). Propanoic (CID_1032) and isobutyric (CID_6590) acids have hydrophobic and Pi-stacking interactions (**C**,**D**). Valeric (CID_7991) and isovaleric (CID_10430) acids exhibit H-bond, hydrophobic, Pi-Sigma and Pi-Alkyl interactions (**E**,**F**).

Lpp-pheromones/volatiles interactions were assessed by the docking approach. The large binding volume of Lpp was selected from the binding site analysis. The results showed several H-bond and hydrophobic interactions (Figure 7A–F).

The docking results revealed that the six pheromone compounds showed good binding affinity towards the Lpp (Supplementary File S1 Table S3). Particularly, valeric and isovaleric acids had more binding energy scores with the Lpp compared to all other compounds. Valeric acid depicted very significant H-bonds (Y64, H83, N199), hydrophobic (F81, Q82, E107, E136, N201, N227), and Pi-Sigma (Y86) interactions, respectively. Further, isovaleric acid interacted to an extent with the protein, including H-bond (N227), hydrophobic (H38, F81, Q82, H83, M109, N199, S200, N201), and Pi-Alkyl interaction (Y64). On the other hand, the acetic acid had higher binding frequency (75%) with two H-bond (K142, K221). Similarly, propanoic acid had abundant binding frequency (72%) with Pi-Alkyl interactions. However, valeric acid had a high ligand interaction pattern according to the estimation of binding free energy (−3.16 kcal/mol) with Lpp.

## 4. Discussion

One of the most prevalent bacteria in buffalo CM during the estrus phase is *S. pasteuri* [9]. This study aimed at finding the importance of *S. pasteuri* specifically occurring during estrus, in chemical communication. Therefore, the putative exoproteome of *S. pasteuri* isolated from CM were analyzed. We identified 219 proteins to constitute the *S. pasteuri* exoproteome. Protein synthesis elongation factors (EF-Tu, EF-G) and intracellular chaperones (Hsp60/GroEL, Hsp70/DnaK) are involved in the bacterial adhesion properties [31]. In concordance with these reports, the present results suggest that *S. pasteuri* produces abundant surface proteins that regulate physical interactions with the host cells and tissues. The expression of heat shock proteins (HSPs) was increased in buffalo CM during several physiological and environmental stresses [32]. Presently, Cpn60 belonging to the family of heat shock proteins has been identified which has a role in protecting proteins from denaturation during stress conditions [33]. Cpn60 molecules also play roles as intercellular signalling proteins and can also modulate immune response by stimulating myeloid cell cytokine synthesis. It was proposed that surface expression of Cpn60 acts as an adhesion molecule which would enable the bacteria to effectively bind and interact with host cells [34].

Previously, the protein profile of buffalo CM has been established with respect to the estrous cycle, and a list of estrus-specific proteins has been reported among which enolase is one of the major components [32]. In the present study, enolase has been identified in the exoproteome of *S. pasteuri* isolated from buffalo CM specifically at the estrus phase. Enoyl acyl-carrier protein reductase (ENR) has been reported as a catalyst during fatty acids synthesis [35]. The presence of ENR in the exoproteome of *S. pasteuri* may be associated with the synthesis of volatile fatty acids/pheromones. Additionally, it is established that most of the pheromones reported in mammals during the estrus phase are derived from fatty acids [36,37]. Our study on buffalo CM confirmed that the short chain fatty acids may also act as pheromones [9]. These identified proteins are largely associated with estrus-related function (s) and may play significant role during the estrus, especially in chemical communication.

Comparative gene ontology analysis revealed that the proteins identified are highly associated with binding properties and regulatory functions. The extracellular proteins in saliva of women are involved in essential roles during ovulation [38]. It is to be noted that proteins having binding properties are being identified in body fluids along with the volatiles, which facilitate chemical communication during the estrus phase of mammals [39,40]. Also, a report showed a large number of binding proteins in saliva during the estrus phase of buffalo compared to the other phases [41]. These results suggest that the binding proteins may be engaged in a few essential shuttle roles of pheromone communication during the estrus phase. In addition, the binding proteins might be involved in increasing the stability of other co-expressing proteins [41].

The in silico modelled Lpp residues were used to crosscheck the protein-ligand interaction. The 3D-structural model of Lpp has close evolutionary relationship with 4EF1 and shows several conserved residues with the typical β-barrel structures, and alpha-helix with interconnected loops. The Lpp structure and biosynthetic pathways have been investigated in *E. coli* [42,43]. The modelled Lpp possesses multiple binding sites, which are preferentially involved in accommodating multiple odor/volatile molecules [42]. The mesh and solid surface view of Lpp facilitated the understanding of the surface area and volume of the protein, where small molecules may be recruited to enter the inactive site of the protein to enhance the residual interaction with the receptor protein [44].

Previously we have reported that estrus-specific *S. pasteuri* may produce volatiles such as acetic-, butanoic-, propanoic-, isobutyric-, valeric-, and isovaleric acids [9]. Eventually, the present docking analysis has shown that the volatiles/pheromones have significant H-bonding and hydrophobic interactions with the Lpp binding pocket. The presence of salt bridges, hydrophobic contacts and hydrogen bonds may help to improve the binding efficiency during protein-pheromone interactions. The previous reports on buffalo nasal OBP [17,18] and estrus urinary lipocalin protein in rat [45,46] have shown binding affinity with volatiles/ligands in regard to pheromone/chemical communication in mammals. The identified Lpp is closely similar to *Enterococcus faecalis V583* pheromone cOB1 precursor/lipoprotein. However, the phylogenetic tree revealed the lipoprotein of estrus-specific *S. pasteuri* as closely related to lipocalin family proteins (aphrodisin, bovine OBP and bunOBP). This suggests that *S. pasteuri* Lpp may also be a pheromone carrier, like major urinary proteins in rat [45,46] and aphrodisin (major soluble protein belonging to lipocalin) in hamster vaginal secretion [47,48]. Also, a study demonstrated that this lipocalin functions as a volatile pheromone-carrier protein, in addition to its own role as pheromone in the vaginal secretion of mouse [49]. The highly expressed lipocalin-2 (LCN2) during estrus is involved in the interaction between symbiotic bacteria and the host [49]. It is known that during metabolic degradation in most mucosal tissues, bacteria attempt to acquire ‘free’ iron by a secretion of high-affinity iron sequestrating siderophores. The mammalian host, however, limits this process by the production of lipocalin 2 [50] which efficiently scavenges catecholate-type siderophores [51]. Thus, bacterial Lpp represents an efficient regulatory element and may play a supporting role as a pheromone shuttle during estrus in the buffalo vaginal secretion.

Altogether, the existence of Lpp in the *S. pasteuri* exoproteome suggests a probable involvement of Lpp in pheromone-binding by acting as a pheromone carrier during the buffalo estrus phase. However, more focussed studies are required to establish estrus-specific bacterial Lpp as a pheromone-carrier protein and its significance in chemical communication of buffalo with regard to inter-kingdom (bacteria-host animal) communication.

## Figures and Tables

**Table 1:** List of functionally important proteins identified from culture supernatant of estrus-specific *S. pasteuri*.

UniProt ID	Protein Names	Function ^a^	#Peptides	#AAs Length	MW [kDa]	Calc. pI
A0A0M2NQR9	Elongation factor Tu (EF-Tu)	Translation elongation factor activity	22	394	43.2	4.87
A0A431ZL22	60 kDa chaperonin (Cpn60)	ATP binding	14	541	57.5	4.67
A0A269XJ15	Enolase	Magnesium ion binding	13	434	47.2	4.65
A0A269XHD8	Fructose-bisphosphate aldolase class 1	Fructose-bisphosphate aldolase activity	12	296	33	4.91
A0A0M2NSM8	Lipoprotein (Lpp)	Lipid binding protein	2	269	30.1	9.26

^a^ Functions were retrieved using the ComparativeGo online database bioinformatics resource.

## Data Availability

The data is available on reasonable request from the corresponding author.

## Supplementary Materials

The following supporting information can be downloaded at: https://www.mdpi.com/article/10.3390/biom12030450/s1, Supplementary File S1, Supplementary File S2, Supplementary File S3. Figure S1: Growth curve of *S. pasteuri*; Table S1: Physiological properties of identified compounds from bacteria *S. pasteuri* of estrus CM; Figure S2: SDS-PAGE; Table S2: List of total proteins identified from *S. pasteuri* secretome; Figure S3: Tandem mass spectrum of Lpp; Figure S4: Gene Ontology; Figure S5: The secondary structure prediction of Lpp using SOPMA and ProFunc; Table S3: The Lpp-compounds/ligand interactions.
