# Supplementary material for: The Exoproteome of Staphylococcus pasteuri Isolated from Cervical Mucus during the Estrus Phase in Water Buffalo (Bubalus bubalis)
Source: Biomolecules. 2022 Mar 15;12(3):450. doi: 10.3390/biom12030450 (PMC8946806; doi:10.3390/biom12030450)
Supplement: Supplementary file 1 [file biomolecules-12-00450-s001.zip › Supplementary File S1.pdf]

S2.1. Isolation and characterization of bacteria

For the isolation of bacteria, the CM was collected from twelve healthy buffaloes. The CM of all 12 subjects, each running through the three phases of the oestrous cycle, were collected. Thus, we had 36 CM samples (Preestrus 12, Estrus 12, and Diestrus 12). These samples were pooled as samples from four animals representing each phase (Preestrus-4, Estrus-4 and Diestrus-4; thus, four pooled samples for each phase) and subjected to further analysis. Each analysis was conducted on triplicate samples from each pool. CM, 0.5 mL, was homogenized in 4.5 mL of sterile distilled water. An aliquot of 0.1 mL of the homogenate was serially diluted and plated in Mannitol salt- agar by standard plate count method (Cassoli et al. 2016). Plates were incubated at 37°C for 24-48 hr. Following the incubation, the bacterial colonies were counted and expressed by log values (Log CFU/mL i.e. Log10 (CFU / (dilution factor x aliquot). Morphologically distinct colonies alone were picked and further processed. Primary characterization was carried out based on colony morphology which included colour, size and shape. Further, the isolates were confirmed by Gram staining and biochemical tests for catalase, oxidase, sugar fermentation and haemolysis (Srinivasan et al., 2021). Finally the sequence were submitted in NCBI (accession number: MT598013.1).

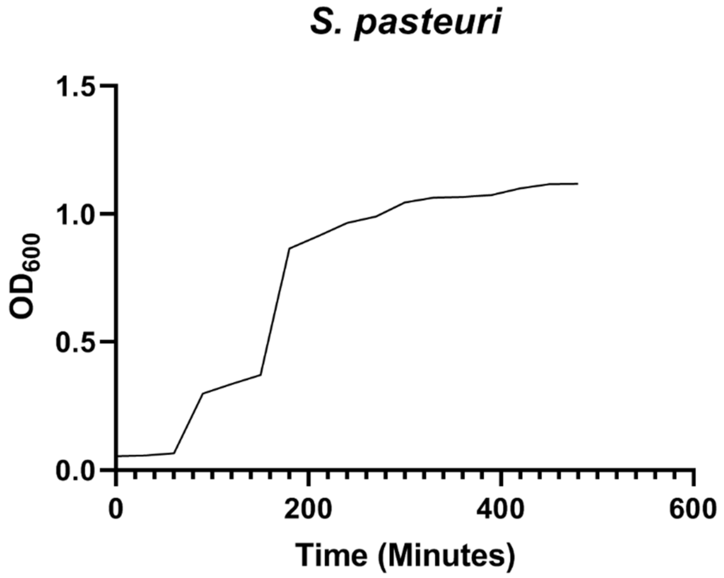

Figure S1:Growth curve of *S. pasteuri*. The OD values are observed at every 30 minutes and the values are plotted for 8 hours.

Table S1. Physiological properties of identified compounds from bacteria *S. pasteuri* of estrus CM

Identified compounds	PubChem	MW (g/mol)	Chemical formula	Canonical smiles
**Acetic acid**	176	60.05	$C_2H_4O_2$	CC(=O)O
**Butanoic acid**	264	88.11	$C_4H_8O_2$	CCCC(=O)O
**Propanoic acid**	1032	74.08	$C_3H_6O_2$	CCC(=O)O
**Isobutyric acid**	6590	88.11	$C_4H_8O_2$	CC(C)C(=O)O
**Valeric acid**	7991	102.13	$C_5H_{10}O_2$	CCCCC(=O)O
**Isovaleric acid**	10430	102.13	$C_5H_{10}O_2$	CC(C)CC(=O)O

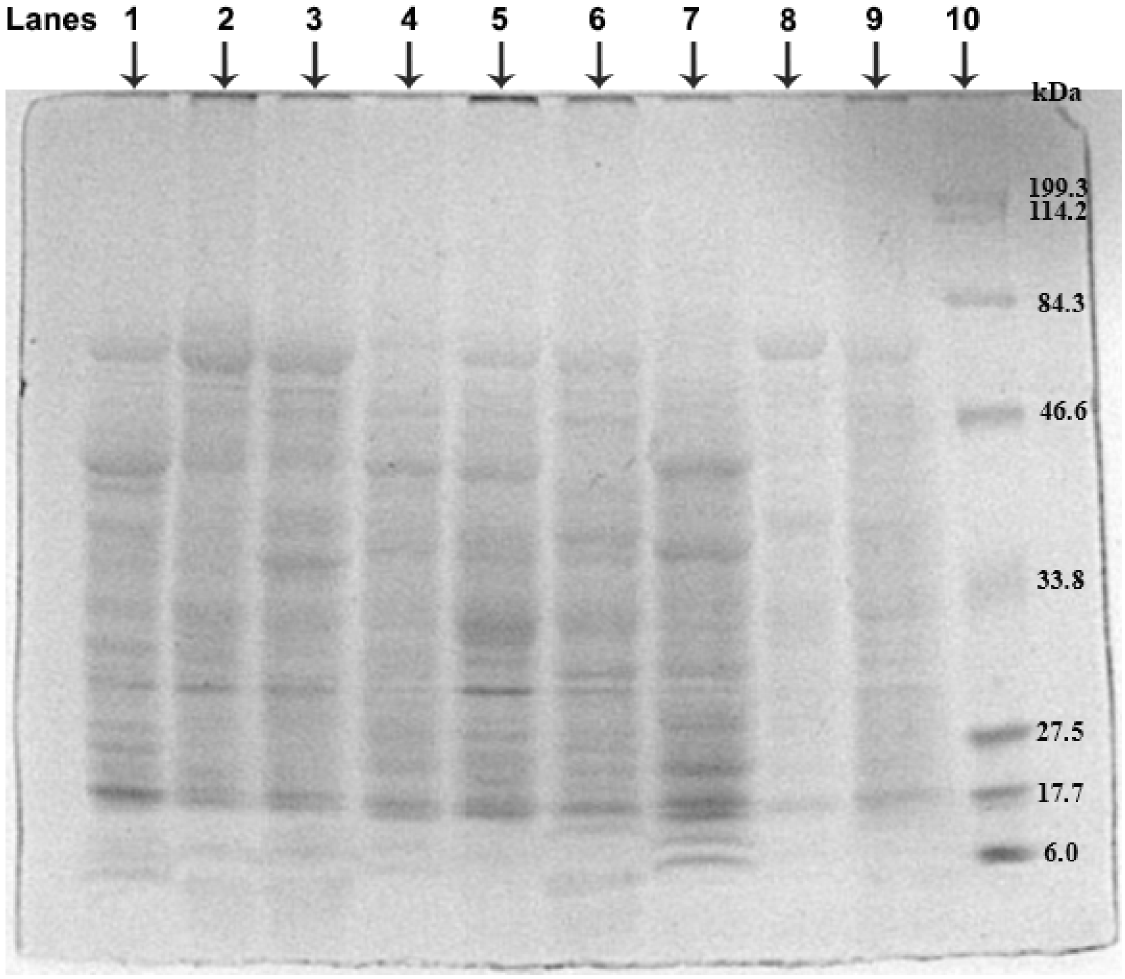

Figure S2. SDS-PAGE. 1D gel electrophoresis of *S. pasteuri* secretory proteins collected during estrus CM. Each lanes have duplicated samples of *S. pasteuri* extracellular proteins (at various concentrations, as follows: Lanes 1-3&5: 20µg; Lane 4, 8&9: 10µg; Lane 6&7: 30µg) along with standard protein marker (Lane 10: 6µL).

Table S2. List of total proteins identified from *S. pasteuri* secretome.

S.No	Protein Accession ID	Protein Description	# AAs	MW [kDa]	calc. pI	# Peptides
**1.**	A0A0M2NQR9	Elongation factor Tu	394	43.2	4.87	22
**2.**	A0A431ZL22	60 kDa chaperonin	541	57.5	4.67	14
**3.**	A0A269XJ15	Enolase	434	47.2	4.65	13
**4.**	A0A269XHD8	Fructose-bisphosphate aldolase class 1	296	33	4.91	12
**5.**	A0A431ZQ83	Elongation factor Tu	396	43.3	4.83	10
**6.**	A0A269XJ26	Elongation factor G	693	76.7	4.89	10
**7.**	A0A269XLM5	Dihydrolipoyl dehydrogenase	468	49.6	4.82	9
**8.**	A0A269XNB0	30S ribosomal protein S4	200	23.2	9.88	9
**9.**	A0A0M2NRP5	ATP synthase subunit beta	470	51.4	4.73	8
**10.**	A0A431ZNX2	ATP synthase subunit beta	471	51.3	4.98	8
**11.**	A0A0M2NQC0	Pyruvate kinase	585	63.1	5.19	8
**12.**	A0A0M2NQR2	DNA-directed RNA polymerase subunit beta	1207	135	6.3	8
**13.**	A0A0M2NUT6	Dihydrolipoyl dehydrogenase	468	49.6	4.87	7
**14.**	A0A269XK66	Glutamate--tRNA ligase	484	56.2	5.07	7
**15.**	A0A0M2NPE4	Glucose-6-phosphate isomerase	443	49.6	5.08	7
**16.**	A0A0M2NJY7	Arginine deiminase	411	47.3	5.17	7
**17.**	A0A269XK51	Dihydrolipoamide acetyltransferase component of pyruvate dehydrogenase complex	435	47	5	7

**18.**	A0A269XIL3	Arginine--tRNA ligase	553	62.6	5.29	6
**19.**	A0A3S0MQB3	Acetolactate synthase AlsS	554	60.5	4.98	6
**20.**	A0A269XH97	Probable malate:quinone oxidoreductase	499	56.2	6.55	6
**21.**	A0A0M2NTM6	Glycerol-3-phosphate dehydrogenase	557	62.3	6.77	6
**22.**	A0A269XJK1	Glyceraldehyde-3-phosphate dehydrogenase	335	36.1	4.97	6
**23.**	A0A0M2NK15	60 kDa chaperonin	540	57.7	4.64	6
**24.**	A0A269XKU5	2,3-bisphosphoglycerate-independent phosphoglycerate mutase	505	56.3	4.86	6
**25.**	A0A269XK85	50S ribosomal protein L2	277	30.2	10.9	5
**26.**	A0A269XHG3	Acetolactate synthase large subunit (Fragment)	482	52.8	4.96	5
**27.**	A0A269XHW6	50S ribosomal protein L5	179	20.2	9.32	5
**28.**	A0A269XH42	L-lactate dehydrogenase	318	34.3	5.16	5
**29.**	A0A269XIT4	Betaine-aldehyde dehydrogenase	496	54.6	4.93	5
**30.**	A0A269XEX6	Formate acetyltransferase	748	84.8	5.48	5
**31.**	A0A0M2NV34	ATP-dependent Clp protease ATP-binding subunit ClpC	817	91	5.68	5
**32.**	A0A0M2NRP2	DNA-directed RNA polymerase subunit beta	1183	132.9	4.97	5
**33.**	A0A0M2NN96	Iron-sulfur cluster assembly protein SufB	465	52.5	5.17	5
**34.**	A0A0M2NR05	Chaperone protein DnaK	611	66.3	4.73	5
**35.**	A0A0M2NR25	Oleate hydratase	591	67.6	6.39	5
**36.**	A0A0M2NX32	Alanine dehydrogenase	372	40	5.35	4
**37.**	A0A3S0LXA1	Alanine dehydrogenase	370	39.8	5.45	4
**38.**	A0A3S0ML13	Enolase	434	47.1	4.61	4

**39.**	A0A431ZL71	Dihydrolipoyl dehydrogenase	468	49	5.16	4
**40.**	A0A0M2NV81	Elongation factor Ts	292	32.3	5.12	4
**41.**	A0A0M2NMB1	ATP synthase subunit alpha	502	54.5	4.92	4
**42.**	A0A0M2NRL0	Uncharacterized protein	199	22.6	5.35	4
**43.**	A0A0M2NRE1	50S ribosomal protein L6	178	19.6	9.35	4
**44.**	A0A3S0M1J9	Chaperone protein DnaK	611	65.9	4.67	4
**45.**	A0A0M2NP59	Translation initiation factor IF-2	724	80	5.17	4
**46.**	A0A269XJN9	1,4-dihydroxy-2-naphthoyl-CoA synthase	272	30.2	5.45	4
**47.**	A0A431ZSJ1	Glucose-6-phosphate isomerase	445	49.7	4.94	4
**48.**	A0A0M2NU72	2,3-bisphosphoglycerate-independent phosphoglycerate mutase	505	56.3	4.89	4
**49.**	A0A0M2NP53	Ribosome-recycling factor	184	20.3	5.07	4
**50.**	A0A0M2NVX0	6-phosphogluconate dehydrogenase, decarboxylating	469	51.9	5.08	4
**51.**	A0A0M2NR12	Lysine--tRNA ligase	495	57	5.29	4
**52.**	A0A0M2NTH8	30S ribosomal protein S2	262	29.7	5.68	4
**53.**	A0A0M2NUC7	Superoxide dismutase	199	22.6	5.11	3
**54.**	A0A431ZD84	Succinate dehydrogenase flavoprotein subunit	587	65.4	5.85	3
**55.**	A0A0M2NS92	Alkyl hydroperoxide reductase C	189	21.1	4.78	3
**56.**	A0A0M2NXE7	Pyridoxal 5'-phosphate synthase subunit PdxS	295	31.9	5.36	3
**57.**	A0A0M2NKL3	GMP synthase [glutamine-hydrolyzing]	513	58	4.92	3
**58.**	A0A431ZQL6	Elongation factor G	693	76.8	4.97	3
**59.**	A0A269XKH3	Alpha-ketoacid dehydrogenase subunit beta	325	35.2	4.73	3

**60.**	A0A3S0MQV9	Glycerol-3-phosphate dehydrogenase	558	62.9	7.28	3
**61.**	A0A0M2NVE2	Phosphoenolpyruvate-protein phosphotransferase	572	63.2	4.69	3
**62.**	A0A0M2NT47	30S ribosomal protein S3	218	24.3	9.72	3
**63.**	A0A0M2NK92	Beta sliding clamp	377	42	4.83	3
**64.**	A0A431ZLM1	Phosphoenolpyruvate-protein phosphotransferase	570	63.1	4.78	3
**65.**	A0A431ZFK4	Enoyl-[acyl-carrier-protein] reductase [NADPH]	257	28.4	6.55	3
**66.**	A0A0M2NP47	ATP-dependent protease ATPase subunit HslU	467	52.5	5.11	3
**67.**	A0A269XG65	Phosphoglucosamine mutase	451	49	4.7	3
**68.**	A0A0M2NUU4	UPF0637 protein UF70_0699	204	24.1	6.92	3
**69.**	A0A0M2NV72	Succinate--CoA ligase [ADP-forming] subunit beta	388	42.1	4.84	3
**70.**	A0A269XKW4	Asparagine--tRNA ligase	430	49.3	5.4	3
**71.**	A0A0M2NQA4	Translation initiation factor IF-3	175	20.3	9.66	3
**72.**	A0A0M2NVT8	GTP-sensing transcriptional pleiotropic repressor CodY	257	28.7	5.95	3
**73.**	A0A0M2NLN7	Inosine-5'-monophosphate dehydrogenase	488	52.5	5.33	3
**74.**	A0A431ZII9	Fe-S cluster assembly ATPase SufC	259	29.2	4.7	2
**75.**	A0A0M2NPX7	Cell division protein FtsZ	392	41.1	4.94	2
**76.**	A0A0M2NNQ3	Cysteine synthase	310	33.1	5.36	2
**77.**	A0A3S0KQH4	Adenylosuccinatelyase	431	49.8	5.76	2
**78.**	A0A0M2NJB2	50S ribosomal protein L25	221	24.4	4.27	2
**79.**	A0A0M2NPS6	GTPase Der	436	49	5.36	2
**80.**	A0A0M2NV51	Dihydroxyacetone kinase family protein	549	60.1	4.64	2

**81.**	A0A0M2NV50	Chaperone protein ClpB	868	98.1	5	2
**82.**	A0A3S0K7L9	Probable malate:quinone oxidoreductase	498	56.3	6.55	2
**83.**	A0A3S0MTQ0	Glyceraldehyde-3-phosphate dehydrogenase	336	36.4	4.93	2
**84.**	A0A0M2NUK8	Oligoendopeptidase F	602	69.8	4.93	2
**85.**	A0A431ZIA0	RNA polymerase sigma factor SigA	369	42.3	5.14	2
**86.**	A0A0M2NV39	Cysteine--tRNA ligase	466	54	5.21	2
**87.**	A0A0M2NUI1	Argininosuccinate synthase	400	44.5	5.01	2
**88.**	A0A0M2NU61	ATP-dependent Clp protease proteolytic subunit	196	21.6	5.03	2
**89.**	A0A0M2NQT1	Glucose-6-phosphate 1-dehydrogenase	494	56.9	5.76	2
**90.**	A0A0M2NVG1	Aconitate hydratase	901	99.2	4.87	2
**91.**	A0A269XJC2	Dihydroxyacetone kinase subunit DhaK	322	35	4.98	2
**92.**	A0A3S0THC8	Beta sliding clamp	377	42.2	4.87	2
**93.**	A0A0M2NX25	ATP-dependent 6-phosphofructokinase	322	34.8	5.58	2
**94.**	A0A3S0K8S3	4-hydroxy-3-methylbut-2-en-1-yl diphosphate synthase (flavodoxin)	366	39.4	5.92	2
**95.**	A0A0M2NUP3	Threonine--tRNA ligase	645	74.7	5.27	2
**96.**	A0A0M2NNA6	Uracil phosphoribosyltransferase	209	23.1	5.9	2
**97.**	A0A269XGV9	Pantothenatesynthetase	283	32	4.93	2
**98.**	A0A3S0MZI0	Glycerophosphoryldiester phosphodiesterase	247	27.9	5.02	2
**99.**	A0A3S0TG30	HPr kinase/phosphorylase	310	34.5	6.44	2
**100.**	A0A269XHX4	Iron citrate ABC transporter substrate-binding protein	335	37.3	9.07	2
**101.**	A0A3S0M671	Glutamine synthetase	446	50.7	5.02	2

**102.**	A0A0M2NP92	Glutamine synthetase	446	50.9	5.12	2
**103.**	A0A0M2NSM8	Lipoprotein	269	30.1	9.26	2
**104.**	A0A3S0KCT6	Ribosome-recycling factor	184	20.5	4.98	2
**105.**	A0A0M2NL96	Two-component response regulator SA14-24	233	27.1	5.12	2
**106.**	A0A0M2NRM8	Ornithine carbamoyltransferase	335	37.7	5.26	2
**107.**	A0A431ZT99	TIGR00282 family metallophosphoesterase	263	29.5	7.2	2
**108.**	A0A3S0NDB3	Purine nucleoside phosphorylase DeoD-type	236	25.6	4.74	1
**109.**	A0A0M2NPD4	Peptidyl-prolyl cis-transisomerase	197	21.7	4.7	1
**110.**	A0A0M2NSM3	Glycine cleavage system H protein	126	14.2	3.94	1
**111.**	A0A431ZQL1	Ornithine aminotransferase	396	43.6	5.24	1
**112.**	A0A431ZS97	DNA-directed RNA polymerase subunit alpha	314	35	4.83	1
**113.**	A0A0M2NM54	Iron-sulfur cluster carrier protein	354	38.5	5.16	1
**114.**	A0A0M2NRC0	1-acyl-sn-glycerol-3-phosphate acyltransferase	205	22.8	9.61	1
**115.**	A0A269XLK0	Tyrosine--tRNA ligase	420	47.5	5.55	1
**116.**	A0A0M2NML5	L-lactate permease	533	56.7	9.16	1
**117.**	A0A0M2NR92	D-serine/D-alanine/glycine transporter	453	50.2	9.42	1
**118.**	A0A0M2NVW7	Protein RecA	348	37.8	5.45	1
**119.**	A0A0M2NTD5	CTP synthase	535	59.9	5.35	1
**120.**	A0A3S0KQ60	Protein translocase subunit SecA	843	96.3	5.33	1
**121.**	A0A3S0M2Q9	Serine hydroxymethyltransferase	413	45.4	5.54	1
**122.**	A0A0M2NR73	ATP-dependent Clp protease ATP-binding subunit ClpX	420	46.3	4.58	1

**123.**	A0A0M2NS80	Hydroxymethylglutaryl-CoA synthase	388	43.3	4.88	1
**124.**	A0A0M2NVV7	Ribonuclease J	557	62.4	6.15	1
**125.**	A0A269XI07	Alkaline shock protein 23	173	19.6	5.06	1
**126.**	A0A3S0KF36	50S ribosomal protein L2	277	30.3	10.84	1
**127.**	A0A0M2NP63	Polyribonucleotide nucleotidyltransferase	699	77.1	5	1
**128.**	A0A269XLL2	UDP-N-acetylmuramate--L-alanine ligase	437	49.4	5.15	1
**129.**	A0A0M2NUH7	Glutamate dehydrogenase	414	45.6	5.17	1
**130.**	A0A0M2NMR4	Uncharacterized protein	205	23.7	4.79	1
**131.**	A0A0M2NQS1	Glutamate-1-semialdehyde 2,1-aminomutase	429	46.9	5.22	1
**132.**	A0A0M2NUS2	Phosphoglycerate kinase	396	42.7	5.08	1
**133.**	A0A0M2NUM8	Glutamate-1-semialdehyde 2,1-aminomutase	428	46.4	5.08	1
**134.**	A0A269XG97	Transglycosylase	246	25.7	5.39	1
**135.**	A0A0M2NM89	Fructose-1,6-bisphosphate aldolase	286	30.7	4.97	1
**136.**	A0A0M2NMH3	3-hexulose-6-phosphate synthase	210	22.5	4.6	1
**137.**	A0A0M2NN17	30S ribosomal protein S5	166	17.7	9.99	1
**138.**	A0A3S0KF75	Protein RecA	360	39	5.87	1
**139.**	A0A269XKD8	Flavohemoglobin	381	42.8	5.06	1
**140.**	A0A0M2NSP9	Uncharacterized protein	258	26.8	6.52	1
**141.**	A0A3S0KKL9	Translation initiation factor IF-3	176	20.4	9.63	1
**142.**	A0A269XMH7	Probable quinol oxidase subunit 1	662	75.1	7.46	1
**143.**	A0A0M2NP49	Two component transcriptional regulator VraR	209	23.5	5.07	1

**144.**	A0A0M2NS09	Acyl-CoA synthetase	529	59.8	5.22	1
**145.**	A0A0M2NQ19	tRNA-t(6)A37 methylthiotransferase	448	51.1	5.52	1
**146.**	A0A269XL77	Peptide methionine sulfoxide reductase MsrA	175	20.3	5.73	1
**147.**	A0A269XJ58	Histidine phosphatase family protein	196	22.3	6.37	1
**148.**	A0A3S0M5J8	Malate dehydrogenase	310	33.3	4.83	1
**149.**	A0A269XGN1	2,3-bisphosphoglycerate-dependent phosphoglycerate mutase	228	26.7	5.66	1
**150.**	A0A0M2NVU3	DNA-binding protein HBsu	90	9.6	9.52	1
**151.**	A0A269XLH9	Acetate kinase	412	45.7	5.66	1
**152.**	A0A269XHG9	50S ribosomal protein L4	207	22.5	10.07	1
**153.**	A0A0M2NWX8	GTPaseObg	430	47	5.36	1
**154.**	A0A0M2NWN4	S-adenosylmethionine synthase	399	44	4.89	1
**155.**	A0A431ZH60	1-deoxy-D-xylulose 5-phosphate reductoisomerase	376	41.9	5.27	1
**156.**	A0A0M2NV13	Cell division protein FtsA	465	52.2	4.51	1
**157.**	A0A3S0KC93	Thioredoxin reductase	315	34.1	4.87	1
**158.**	A0A0M2NPY9	Dihydroorotase	425	46.7	6.64	1
**159.**	A0A0M2NUM4	Ribonucleoside-diphosphate reductase	701	80.2	5.4	1
**160.**	A0A0M2NRY5	DNA gyrase subunit A	894	100.4	4.98	1
**161.**	A0A0M2NU79	Phosphate acetyltransferase	329	35.1	4.72	1
**162.**	A0A0M2NSH0	Triosephosphate isomerase	253	27.3	4.88	1
**163.**	A0A0M2NJ35	ThiJ/PfpI family protein	172	18.6	4.63	1
**164.**	A0A0M2NSC2	Ribonucleoside-diphosphate reductase	324	37.7	5.01	1

**165.**	A0A269XLI1	RNA methyltransferase	380	43.2	5.19	1
**166.**	A0A431ZME9	Alpha,alpha-phosphotrehalase	547	63.7	4.98	1
**167.**	A0A0M2NUL0	S-adenosylmethionine:tRNAribosyltransferase-isomerase	341	38.9	5.4	1
**168.**	A0A0M2NQF9	Chorismate mutase I	363	40.8	6.23	1
**169.**	A0A431ZCX0	Acetate kinase	398	43.7	5.74	1
**170.**	A0A0M2NQT8	DNA ligase	668	75.6	5.22	1
**171.**	A0A0M2NP84	Glycerol uptake facilitator protein	272	28.1	8.25	1
**172.**	A0A0M2NSI7	L-Cystine ABC transporter, periplasmic cystine-binding protein TcyA	260	29	9.48	1
**173.**	A0A0M2NQF4	Bifunctional protein GlmU	454	49.5	6.1	1
**174.**	A0A0M2NT61	Adenylate kinase	216	24.1	4.83	1
**175.**	A0A3S0M4Z6	Molybdenum cofactor biosynthesis protein B	166	18.4	6.7	1
**176.**	A0A0M2NT03	Probable quinol oxidase subunit 2	372	42.5	9.07	1
**177.**	A0A0M2NVJ7	ABC transporter ATP-binding protein uup	533	60.1	4.88	1
**178.**	A0A0M2NJN7	Probable manganese-dependent inorganic pyrophosphatase	309	34.1	4.61	1
**179.**	A0A0M2NQQ9	Glycerol-3-phosphate dehydrogenase [NAD(P)+]	332	35.9	5.57	1
**180.**	A0A3S0KKN2	Isocitrate dehydrogenase [NADP]	422	46.4	5.02	1
**181.**	A0A0M2NRL6	Peptidase M20 domain-containing protein 2	394	42.8	5.69	1
**182.**	A0A3S0KFP5	L-lactate permease	534	56.6	9.14	1
**183.**	A0A3S0MMD7	Betaine-aldehyde dehydrogenase	496	54.7	4.88	1
**184.**	A0A0M2NVB8	Probable quinol oxidase subunit 1	662	75.1	7.28	1
**185.**	A0A0M2NUY5	NERD domain-containing protein	302	35	6.34	1

**186.**	A0A0M2NUY0	Transaldolase	237	25.8	4.83	1
**187.**	A0A3S0THZ0	50S ribosomal protein L1	230	24.9	9.32	1
**188.**	A0A3S0T9M6	M20/M25/M40 family metallo-hydrolase	371	40.1	4.91	1
**189.**	A0A269XGL6	Alpha-ketoacid dehydrogenase subunit beta	346	36.8	4.87	1
**190.**	A0A0M2NRD2	Serine--tRNA ligase	428	48.9	5.06	1
**191.**	A0A0M2NTW2	Bacitracin export permease protein BceB	629	71.7	9.41	1
**192.**	A0A269XHU6	UDP-N-acetylglucosamine 1-carboxyvinyltransferase	421	44.8	5.92	1
**193.**	A0A3S0MQP6	Leucine--tRNA ligase	794	90.9	5.01	1
**194.**	A0A431ZLJ1	Alpha-ketoacid dehydrogenase subunit beta	325	35.5	4.81	1
**195.**	A0A0M2NVK2	2,3,4,5-tetrahydropyridine-2,6-dicarboxylate N-acetyltransferase	239	25.4	5.02	1
**196.**	A0A0M2NM68	Oxidoreductase ylbE	217	23.9	5.02	1
**197.**	A0A0M2NKY0	Putative pyridine nucleotide-disulfide oxidoreductase	453	50.1	4.81	1
**198.**	A0A269XIL4	Alcohol dehydrogenase	345	37.2	4.87	1
**199.**	A0A0M2NQH3	D-alanine aminotransferase	282	32.1	5.11	1
**200.**	A0A431ZSC4	Ribonuclease Y	519	58.7	5.64	1
**201.**	A0A3S0KHN1	Pyrimidine-nucleoside phosphorylase	433	46.4	4.96	1
**202.**	A0A0M2NNN3	Ribosomal RNA small subunit methyltransferase A	296	33.5	5.74	1
**203.**	A0A0M2NWV5	Branched-chain-amino-acid aminotransferase	358	40.3	4.79	1
**204.**	A0A3S0KDR8	Coproheme III oxidative decarboxylase	250	29.3	4.84	1
**205.**	A0A3S0ME43	Glycosyltransferase family 2 protein	304	34.9	5.63	1
**206.**	A0A0M2NTE6	Serine hydroxymethyltransferase	412	45.1	6.3	1

**207.**	A0A431ZMD6	Cell division protein DivIB	265	30	6.38	1
**208.**	A0A269XG39	Glutamine--fructose-6-phosphate aminotransferase [isomerizing]	601	66.2	5.05	1
**209.**	A0A0M2NNJ7	AminoacyltransferaseFemA	411	48.8	8.65	1
**210.**	A0A3S0KC52	DUF1430 domain-containing protein	666	78.6	9.26	1
**211.**	A0A3S0MTT8	DNA polymerase III subunit gamma/tau	563	63.3	6.32	1
**212.**	A0A0M2NW00	Uncharacterized protein	83	10	8.29	1
**213.**	A0A431ZCX8	Alpha-ketoacid dehydrogenase subunit beta	327	35.9	4.77	1
**214.**	A0A431ZMZ2	Lipoyl synthase	306	35.2	8.31	1
**215.**	A0A0M2NMC9	D-alanine--D-alanine ligase	356	40.2	4.94	1
**216.**	A0A0M2NQ23	NAD-specific glutamate dehydrogenase	355	39.2	4.91	1
**217.**	A0A3S0NC57	UPF0365 protein CD129_05785	331	35.2	6.98	1
**218.**	A0A0M2NVP2	Dihydrolipoyllysine-residue succinyltransferase component of 2-oxoglutarate dehydrogenase complex	428	46.9	4.88	1
**219.**	A0A0M2NX74	Putative cell-wall-anchored protein SasC (LPXTG motif)	1862	201.4	4.6	1

A B
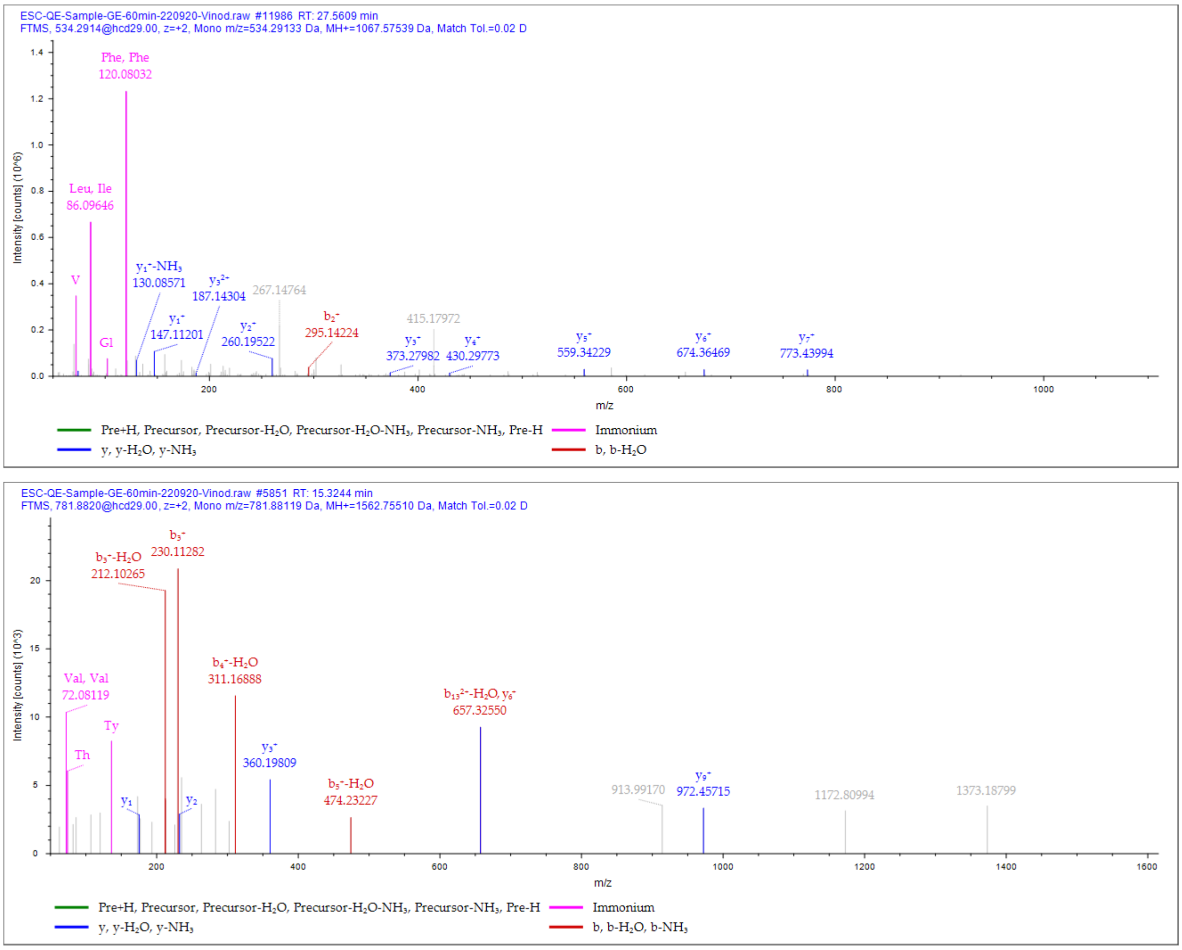

C >A0A0M2NSM8|Lipoprotein OS=*Staphylococcus pasteuri*
MKKILGILLLLTLTVALAACGGKGSDKTIKVGASPAPHAEILEKAKPL
LEKKGYKLDIKTINDYTTPNKLLDKGELDANFFQHTPYLKTESKEKG
YKIESAGNVELEPMAVYSKKYKSLKDLPK**GATVYVSNNPAEQGR**F
LK**FFVDEGLIK**IKKGVKIEDAKFSDITENKKDIKFNNKQSAEFLPKIY
QKQDADAVIINSNYALDQKLNPKKDSIAYEKAKDNPYANLIAVKDGH
KDDKKIKALMEVLQSKEIRDYINKEYDGAVVPAK

Figure S3. Tandem mass spectrum of Lpp. Two representative MS/MS peptide sequences –143–FFVDEGLIK-151 (**A**) and 125-GATVYVSNNPAEQGR-139 (**B**) of Lpp found in the secretome of *S. pasteuri*. Matched representative peptides of Lpp are highlighted in underlined bold red (**C**).

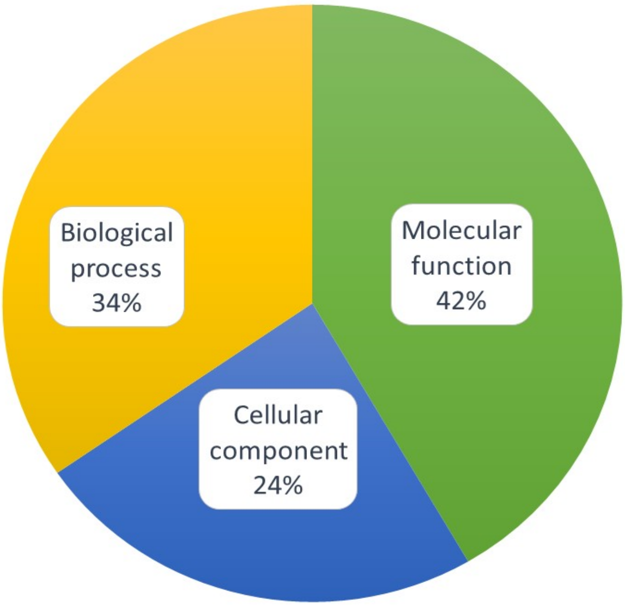

Figure S4. Gene Ontology. Classification of*S. pasteuri* secretory proteins. The majority of the proteins were present in following order, molecular function (42%), biological process (34%) and cellular component (24%) according to GO classification.

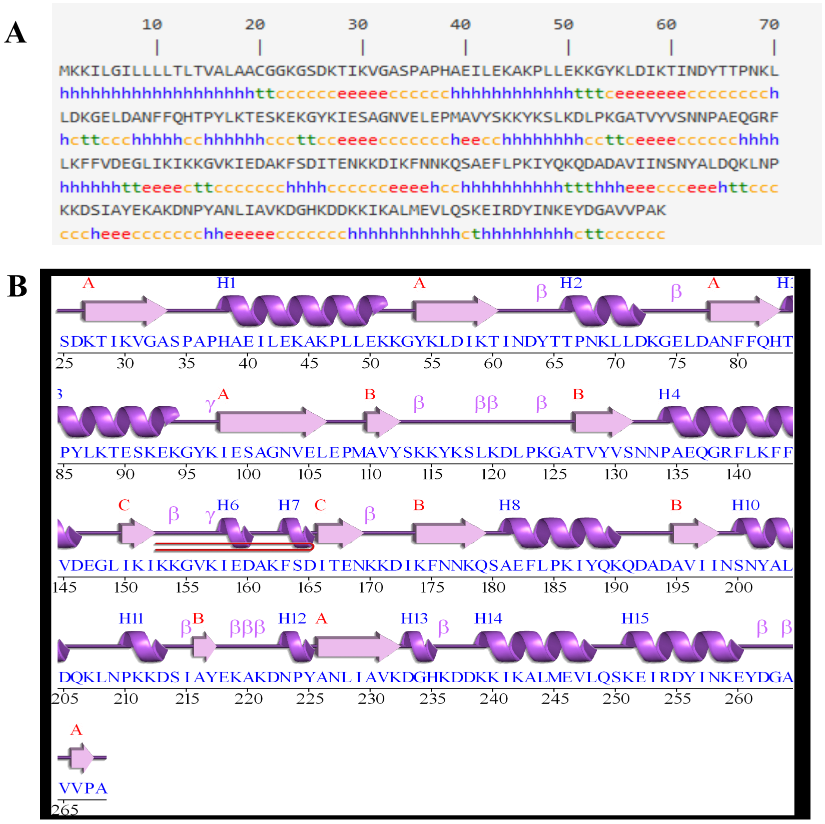

Figure S5. The secondary structure prediction of Lpp using SOPMA and ProFunc. A. The helix (Blue), turn (Green), coil (Purple), and strands (Red) are indicated in SOPMA results. B. The secondary structural components are displayed for the Lpp sequence.

Table S3. The Lpp-compounds/ligand interactions. The values of molecular docking score were retrieved by the best interactions based on dock possess, and Hydrogen bond. The significant Pi-sigma interaction was observed in Valeric acid, and the residues are indicated with bold for Pi-stackinginteractions.

Identified compounds	PubChem	Moldock score	Est. free energy of binding (kcal/mol)	Binding frequency	H-bond	Hydrophobic interactions	Pi-stackinginteractions	Dock posses
**Acetic acid**	176	-26.827	-2.79	75%	K142, K221	P224		1
**Butanoic acid**	264	-26.005	-2.84	36%	K142, K221	I158, E159, P224, Y225		2
**Propanoic acid**	1032	-35.485	-2.85	72%			M109, V130, P140, I197	1
**Isobutyric acid (2-methyl-Propanoic acid)**	6590	-39.43	-3.07	34%		S131, N133, E136, Q137, F140	M109, V130, R139, F163, I197	1
**Valeric acid (2-Methylbutanoic acid)**	7991	-63.079	-3.16	44%	Y64, H83, N199	Q82, E107, E136, N201, N227	**F81, Y86**	2
**Isovaleric acid (3-Methylbutanoic**	10430	-55.061	-3.31	48%	N227	F81, Q82, M109, N199,	H38, Y64, H83	1

**acid)**	S200, N201
