# Supplementary material for: The Exoproteome of Staphylococcus pasteuri Isolated from Cervical Mucus during the Estrus Phase in Water Buffalo (Bubalus bubalis)
Source: Biomolecules. 2022 Mar 15;12(3):450. doi: 10.3390/biom12030450 (PMC8946806; doi:10.3390/biom12030450)
Supplement: Supplementary file 1 [file biomolecules-12-00450-s001.zip › Supplementary File S2.pdf]

Method of Q Exactive

OVERALL METHOD SETTINGS

Global Settings

Use lock masses	best
Lock mass injection	Full MS
Chrom. peak width (FWHM)	6 s
Time Method duration	60.00 min
Customized Tolerances (+/-)	
Lock Masses	—
Inclusion	—
Exclusion	—
Neutral Loss	—
Mass Tags	—
Dynamic Exclusion	—

Experiment

FULL MS / DD-MS² (TOPN)

General

Runtime	0 to 60 min
Polarity	positive
In-source CID	0.0 eV
Default charge state	2
Inclusion	—
Exclusion	—
Tags	—

Full MS

Microscans	1
Resolution	70,000
AGC target	3e6

Maximum IT	100 ms
Number of scan ranges	1
Scan range	400 to 1750 m/z
Spectrum data type	Profile
dd-MS² / dd-SIM	
Microscans	1
Resolution	17,500
AGC target	1e5
Maximum IT	100 ms
Loop count	10
MSX count	1
TopN	10
Isolation window	2.0 m/z
Isolation offset	0.0 m/z
Scan range	200 to 2000 m/z
Fixed first mass	50.0 m/z
(N)CE / stepped (N)CE	nce: 29
Spectrum data type	Profile
dd Settings	
Minimum AGC target	1.00e3
Intensity threshold	1.0e4
Apex trigger	—
Charge exclusion	unassigned, 1, 7, 8, >8
Multiple charge states	all
Peptide match	preferred
Exclude isotopes	on
Dynamic exclusion	15.0 s
If idle ..	do not pick others

Setup

TUNEFILES

General

Switch Count 0

Base Tunefile C:\Xcalibur\Tune files\ESC-QE-nanotune-180418.mstune

CONTACT CLOSURE

General

Used True

Start in Closed False

Switch Count 0

SYRINGE

General

Used False

Start in OFF True

Stop at end of run False

Switch Count 0

Pump setup

Syringe type Hamilton

Flow rate 3.000 µL/min

Inner diameter 2.303 mm

Volume 250 µL

DIVERT VALVE A

General Used False

Start in 1-2 True

Switch Count 0

DIVERT VALVE B

General

Used False

Start in 1-2 True

Switch Count 0

LOCK MASSES

1 entry Mass Polarity Start End Comment [m/z] [min] [min] 445.12003 Positive

Sample pickup:

Volume [µl] : 18.00

Flow [µl / min] : 5.00

Sample loading:

Volume [µl] : 20.00

Flow [µl / min] : 1.00

Max. pressure [Bar] : 400.00

Gradient:

Time [mm:ss]	Duration [mm:ss]	Flow [nl/min]	Mixture [%B]
00:00	00:00	300	0
35:00	35:00	300	25
48:00	13:00	300	90
54:00	06:00	300	90
60:00	06:00	300	3

Pre-column equilibration:

Volume [µl] : 0.00

Flow [µl / min] : 2.00

Max. pressure [Bar] : 350.00

Analytical column equilibration:

Volume [µl] : 3.00

Flow [µl / min] : 1.00

Max. pressure [Bar] : 400.00

Auto-sampler wash:

Flush volume [µl] : 100.00

=== Tune Data: ===:

Spray Voltage (+): 2200.00

Spray Voltage (-): 1500.00

Capillary Temperature (+ or +-): 305.00

Capillary Temperature (-): 250.00

Sheath Gas (+ or +-): 0.00

Sheath Gas (-): 0.00

Aux Gas (+ or +-): 0.00

Aux Gas (-): 0.00

Spare Gas (+ or +-): 0.00

Spare Gas (-): 0.00

Max Spray Current (+): 50.00

Max Spray Current (-): 50.00

Probe Heater Temp. (+ or +-): 350.00

Probe Heater Temp. (-): 350.00

S-Lens RF Level: 56.00

Ion Source: NSI

=== Calibration Data: ===:

Mass Cal. (+) age (d): 4.90

Mass Cal. (-) age (d): 4.89

Isolation Cal. (+) age (d): 99.1

Isolation Cal. (-) age (d): 99.1

Amplifier Gain: 1000.000

Mass Calibration Parameter (0): 7.90000000e+01

Mass Calibration Parameter (1): 1.00000000e+02

Mass Calibration Parameter (2): 1.73279398e-09

Mass Calibration Parameter (3): 1.59220758e+09

Mass Calibration Parameter (4): -2.30194649e-12

Mass Calibration Parameter (5): 1.59220758e+09

Mass Calibration Parameter (6): -2.66331722e-10

Mass Calibration Parameter (7): 1.55126609e+09

Mass Calibration Parameter (8): 4.92801661e-13

Mass Calibration Parameter (9): 1.55126609e+09

Mass Calibration Parameter (10): -1.14000000e-06

Mass Calibration Parameter (11): 0.00000000e+00

Mass Calibration Parameter (12): -1.14000000e-06

Mass Calibration Parameter (13): 0.00000000e+00

Mass Calibration Parameter (14): -1.02106533e-12

Mass Calibration Parameter (15): 1.59220760e+09

Mass Calibration Parameter (16): -7.54030880e-13

Mass Calibration Parameter (17): 1.55126610e+09

Mass Calibration Parameter (18): -6.40724443e-11

Mass Calibration Parameter (19): 1.43098704e+09

Mass Calibration Parameter (20): -3.11636511e-11

Mass Calibration Parameter (21): 1.39090074e+09

Mass Calibration Parameter (22): 0.00000000e+00

Mass Calibration Parameter (23): 0.00000000e+00

Mass Calibration Parameter (24): 0.00000000e+00

Mass Calibration Parameter (25): 0.00000000e+00

Mass Calibration Parameter (26): 0.00000000e+00

Mass Calibration Parameter (27): 0.00000000e+00

Mass Calibration Parameter (28): 0.00000000e+00

Mass Calibration Parameter (29): 0.00000000e+00

Mass Calibration Parameter (30): 4.20000000e+01

Mass Calibration Parameter (31):	1.00000000e+00
Mass Calibration Parameter (32):	1.00000000e+00
Mass Calibration Parameter (33):	0.00000000e+00
Mass Calibration Parameter (34):	2.57694532e+01
Mass Calibration Parameter (35):	1.17147754e+06
Mass Calibration Parameter (36):	1.17147754e+05
Mass Calibration Parameter (37):	1.28000000e+03
Mass Calibration Parameter (38):	2.56000000e+02
Mass Calibration Parameter (39):	1.60034644e+09
Mass Calibration Parameter (40):	6.00000000e+00
Mass Calibration Parameter (41):	1.38066190e+02
Mass Calibration Parameter (42):	7.00989448e+02
Mass Calibration Parameter (43):	1.95087652e+02
Mass Calibration Parameter (44):	5.89713307e+02
Mass Calibration Parameter (45):	5.24264964e+02
Mass Calibration Parameter (46):	3.59733856e+02
Mass Calibration Parameter (47):	1.22199064e+03
Mass Calibration Parameter (48):	2.35626093e+02
Mass Calibration Parameter (49):	1.42197786e+03
Mass Calibration Parameter (50):	2.18429370e+02
Mass Calibration Parameter (51):	1.62196509e+03
Mass Calibration Parameter (52):	2.04520553e+02
Mass Calibration Parameter (53):	1.00000000e+00
Mass Calibration Parameter (54):	1.00000000e+00
Mass Calibration Parameter (55):	1.00000000e+00
Mass Calibration Parameter (56):	2.57422190e+01
Mass Calibration Parameter (57):	9.76791655e+05
Mass Calibration Parameter (58):	9.76791655e+04
Mass Calibration Parameter (59):	2.40000000e+03
Mass Calibration Parameter (60):	2.56000000e+02
Mass Calibration Parameter (61):	1.60034674e+09

Mass Calibration Parameter (62):	8.00000000e+00
Mass Calibration Parameter (63):	2.65147903e+02
Mass Calibration Parameter (64):	5.05903486e+02
Mass Calibration Parameter (65):	5.14284397e+02
Mass Calibration Parameter (66):	3.63252270e+02
Mass Calibration Parameter (67):	1.27999721e+03
Mass Calibration Parameter (68):	2.30252154e+02
Mass Calibration Parameter (69):	1.37999083e+03
Mass Calibration Parameter (70):	2.21753275e+02
Mass Calibration Parameter (71):	1.47998444e+03
Mass Calibration Parameter (72):	2.14131002e+02
Mass Calibration Parameter (73):	1.57997805e+03
Mass Calibration Parameter (74):	2.07244311e+02
Mass Calibration Parameter (75):	1.67997166e+03
Mass Calibration Parameter (76):	2.00982013e+02
Mass Calibration Parameter (77):	1.77996528e+03
Mass Calibration Parameter (78):	1.95255127e+02
Mass Calibration Parameter (79):	0.00000000e+00
Mass Calibration Parameter (80):	0.00000000e+00
Mass Calibration Parameter (81):	0.00000000e+00
Mass Calibration Parameter (82):	0.00000000e+00
Mass Calibration Parameter (83):	0.00000000e+00
Mass Calibration Parameter (84):	0.00000000e+00
Mass Calibration Parameter (85):	0.00000000e+00
Mass Calibration Parameter (86):	0.00000000e+00
Mass Calibration Parameter (87):	0.00000000e+00
Mass Calibration Parameter (88):	0.00000000e+00
Mass Calibration Parameter (89):	0.00000000e+00
Mass Calibration Parameter (90):	0.00000000e+00
Mass Calibration Parameter (91):	0.00000000e+00
Mass Calibration Parameter (92):	0.00000000e+00

Mass Calibration Parameter (93):	0.00000000e+00
Mass Calibration Parameter (94):	0.00000000e+00
Mass Calibration Parameter (95):	0.00000000e+00
Mass Calibration Parameter (96):	0.00000000e+00
Mass Calibration Parameter (97):	0.00000000e+00
Mass Calibration Parameter (98):	0.00000000e+00
Mass Calibration Parameter (99):	0.00000000e+00
Mass Calibration Parameter (100):	0.00000000e+00
Mass Calibration Parameter (101):	0.00000000e+00
Mass Calibration Parameter (102):	0.00000000e+00
Mass Calibration Parameter (103):	0.00000000e+00
Mass Calibration Parameter (104):	0.00000000e+00
Mass Calibration Parameter (105):	0.00000000e+00
Mass Calibration Parameter (106):	0.00000000e+00
Mass Calibration Parameter (107):	0.00000000e+00
Mass Calibration Parameter (108):	0.00000000e+00
Mass Calibration Parameter (109):	0.00000000e+00
Mass Calibration Parameter (110):	0.00000000e+00
Mass Calibration Parameter (111):	0.00000000e+00
Mass Calibration Parameter (112):	0.00000000e+00
Mass Calibration Parameter (113):	0.00000000e+00
Mass Calibration Parameter (114):	0.00000000e+00
Mass Calibration Parameter (115):	0.00000000e+00
Mass Calibration Parameter (116):	0.00000000e+00
Mass Calibration Parameter (117):	0.00000000e+00
Mass Calibration Parameter (118):	0.00000000e+00
Mass Calibration Parameter (119):	0.00000000e+00

CTCD Scale (+): 2.035

CTCD Scale (-): 3.884

CTCD Para (+): 0.800

CTCD Para (-): 0.500

Detect Delay: 5.50

Relais Delay: 5.00

Res.-Dep. Delay: 10.00

Quad DC (+,0): 3.600

Quad DC (+,1): 0.000

Quad DC (-,0): -4.772

Quad DC (-,1): 0.000

Quad OTK (+,0): -24.983

Quad OTK (+,1): -0.056

Quad OTK (-,0): 13.110

Quad OTK (-,1): 0.077

Quad Para A: 0.000

Quad Para B: 0.000

Quad Para C: 0.000

CLT GND Voltage (+): 21.60

CLT GND Voltage (-): 14.20

CLT Offset Voltage (+): 1650.0

CLT Offset Voltage (-): 1650.0

CLT Push Voltage (+): 110.0

CLT Push Voltage (-): 110.0

CLT Pull Voltage (+): 280.0

CLT Pull Voltage (-): 280.0

Lens 6 Voltage (+): 670.0

Lens 6 Voltage (-): 670.0

Z-Lens 3 Voltage (+): 230.0

Z-Lens 3 Voltage (-): 230.0

De-Inject Voltage (+): 40.0

De-Inject Voltage (-): 40.0

De-Measure Voltage (+): 470.0

De-Measure Voltage (-): 470.0

CE-Inject Voltage (+): 3800.0

CE-Inject Voltage (-): 3800.0

LowSNQuanLevel: 1

=== Configuration Data: ===:

Preamp Protect Mode: 0

TMP Sweep Mode: 1

Temp Sensor Mode: 0

=== Identification: ===:

SW Version: 2.9-290033/2.9.0.2926
